# Chitosan-selenium alleviates AFB1-induced growth impairment and immunotoxicity in broilers by suppressing splenic oxidative stress, inflammation and apoptosis

**DOI:** 10.3389/fvets.2026.1859938

**Published:** 2026-05-29

**Authors:** Shuo Yuan, Mengqi Wang, Yu Du, Yitong Liu, Fuguang Liu, Fu Chen, Xueyuan Hu, Linlin Hou

**Affiliations:** 1 College of Veterinary Medicine, Qingdao Agricultural University, Qingdao, China; 2 Animal Disease Prevention and Control Center of Muping District, Yantai, China

**Keywords:** aflatoxin B1, apoptosis, broiler, chitosan-selenium, immunotoxicity

## Abstract

**Introduction:**

Aflatoxin B1 (AFB1) is a prevalent mycotoxin that severely compromises poultry growth performance and induces immunotoxicity. While selenium is known to mitigate mycotoxicosis, the protective efficacy of chitosan-selenium (Ct-Se), a novel organic formulation, against AFB1-induced splenic immunotoxicity in broilers remains unclear.

**Methods:**

48 one-day-old AA broilers were randomly assigned to four groups: control (CON), AFB1 (0.6 mg/kg), Ct-Se (0.4 mg/kg) and AFB1+Ct-Se groups. Following a 42-day trial, we evaluated growth performance, immune organ indices, oxidative stress, inflammatory cytokines, splenic histopathological lesions and apoptosis.

**Results:**

Ct-Se alleviated AFB1-induced growth impairments by improving body weight, average daily gain, and feed conversion ratio. Furthermore, dietary Ct-Se reversed the AFB1-induced suppression of serum IgG and IgM levels, while elevating SOD, GSH-Px, T-AOC activities, and reducing MDA accumulation. Moreover, Ct-Se mitigated AFB1-triggered inflammatory responses by suppressing the expression of pro-inflammatory cytokines and upregulating anti-inflammatory cytokines. Histopathological analysis revealed that Ct-Se effectively attenuated AFB1-induced splenic injury by alleviating lymphocyte loss, red pulp congestion, and vacuolization. Furthermore, Ct-Se significantly suppressed splenic apoptosis by reversing the AFB1-induced upregulation of Bax, Caspase-3, and Caspase-9, while restoring Bcl-2 expression.

**Discussion:**

Our findings demonstrate that Ct-Se alleviates AFB1-induced growth impairment and splenic immune dysfunction involving antioxidant, anti-inflammatory, and anti-apoptotic mechanisms, suggesting its potential as a protective feed additive against broiler mycotoxicosis.

## 1 Introduction

Aflatoxin (AF) is one of the most common contaminants in feed raw materials, mainly produced by the secondary metabolism of *Aspergillus flavus* and *Aspergillus parasiticus*. Among them, AFB1 is currently known as the most toxic and widespread (1). Even at a low dosage, AFB1 could result in oxidative stress, inflammatory response, immunosuppression, ultimately leading to impaired growth performance in various animals (2–4). AFB1 induced immunotoxicity, characterized by lymphocyte depletion and immune organ dysfunction, thereby severely compromising immune function and threatening poultry health. The immunotoxicity of AFB1 is mainly manifested as reductions in immune index, suppression of T cell activation and antibody production, and depletion of lymphocyte populations (5). Studies have shown that AFB1 exposure significantly reduces the levels of immunoglobulins G (Ig G), IgA, IgM, and to down-regulate key immune cytokines, such as interferon-*γ* (IFN-γ) and tumor necrosis factor alpha (TNF-*α*), and also cause a decrease in the CD4^+^/CD8^+^ ratio (3).

The spleen, as the largest peripheral immune organ, plays a central role in immune surveillance, lymphocyte activation, humoral immune regulation, and has been identified as one of the major immune target organs affected by AFB1 (6, 7). AFB1 exposure could lead to congestion of the red pulp of the spleen, depletion of lymphocytes in the white pulp, and histopathological lesions, such as vacuolation and nuclear fragmentation in splenic follicles and periaortic lymphatic sheaths (8). In addition, AFB1 triggers upregulation of pro-apoptotic factors Bax, and activation of mitochondrial pathway apoptosis, resulting in increased splenic cell apoptosis and consequent impairment of splenic structure and function. Mechanistically, AFB1-induced oxidative stress and inflammatory responses are considered key drivers of splenic immune injury (9), as they disrupt cellular redox homeostasis and activate inflammatory signaling pathways, subsequently upregulating pro-apoptotic proteins and triggering splenocyte apoptosis.

Selenium is an essential trace element for humans and animals, playing a critical role in antioxidant defense and immune function. Supplementation with selenium has been shown to mitigate the immunotoxicity induced by AFB1, as evidenced by enhanced T-cell proliferation, and improved cytokine secretion (10, 11). Studies have demonstrated that selenium alleviates AFB1-induced immune dysfunction by upregulating the expression and activity of glutathione peroxidase (GPX) and other selenoproteins (10), thereby enhancing antioxidant capacity and reducing oxidative damage. In addition, selenium has been reported to decrease the AFB1 triggered pro-apoptotic factors such as caspase-3 and caspase-8 while enhancing anti-apoptotic mechanisms in avian lymphoid organs (12). Chitosan-selenium (Ct-Se) is a novel organic selenium formulation that integrates the biological activity of selenium with the biocompatible polymer chitosan. Compared with inorganic selenium, Ct-Se exhibits better solubility and bioavailability, thereby enhancing selenium utilization and its biological efficacy (13), particularly with the respect to antioxidant, anti-inflammatory, and immune-modulatory properties. Our previous studies have demonstrated that Ct-Se supplementation enhances production performance and antioxidant capacity in laying hens and mice, exhibiting superior biological activity compared with inorganic selenium or chitosan alone (14, 15). Notably, compared with the sodium selenite, Ct-Se supplementation significantly increased plasma SOD activity, delayed-type hypersensitivity responses, serum hemolysin levels, and Con A-induced splenocyte proliferation (14–16). Furthermore, Ct-Se has been demonstrated to have protective effects against zearalenone-induced oxidative and immune damage in mice (15). However, the protective effect of Ct-Se on AFB1-induced immune injury in poultry remains unclear.

Therefore, we hypothesized that Ct-Se exerts a protective effect against AFB1-induced immunotoxicity in the spleen by mitigating oxidative stress, suppressing inflammatory responses, and inhibiting apoptosis. To investigate this hypothesis, an AFB1-induced broiler injury model was established to evaluate the protective efficacy of Ct-Se, thereby providing theoretical support for mycotoxin control strategies in animal husbandry.

## 2 Materials and methods

### 2.1 Animals and experimental design

**Figure 1:**
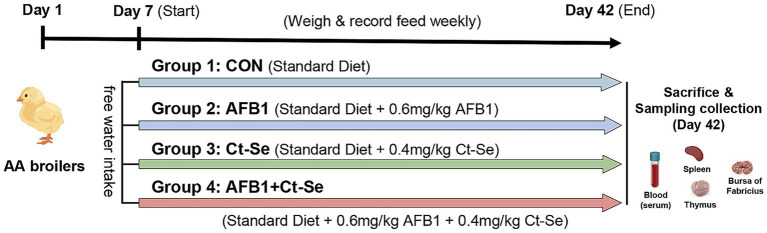
Experimental design, dietary treatments, and sampling schedule of broilers. The 42-day feeding trial started with dietary interventions from Day 7, where broilers were randomly assigned to four groups. The interventions were added directly to the basal diet: AFB1 at a dose of 0.6 mg/kg and Ct-Se at a dose of 0.4 mg/kg with the dose calculated based on selenium content via HPLC-MS. Feed and water were provided *ad libitum*, and growth performance was recorded weekly. At Day 42, broilers were euthanized, and serum, spleen, thymus, and bursa of Fabricius were collected for subsequent analyses.

This study was approved and performed in accordance with the guidelines of the Animal Ethics Committee of Qingdao Agricultural University (Approval No: 2023156). Forty-eight one-day-old male Arbor Acres (AA) broilers were obtained from Yikangbao Breeder Farm (a commercial poultry breeder, Qingdao, China). After a 7-day acclimation period with free access to water and a basal diet, the chicks were randomly divided into four groups with equal numbers: a control group fed the basal diet; an AFB1 group fed basal diet supplemented with 0.6 mg/kg AFB1 (≥98%, HPLC, Shanghai Macklin Biochemical Technology Co., Ltd., China); a Ct-Se group fed basal diet containing 0.4 mg/kg Ct-Se (The Ct-Se used in this study was provided and stored by our laboratory, and calculated based on selenium concentration via HPLC-MS) (15, 16); and a Ct-Se + AFB1 group fed basal diet containing 0.6 mg/kg AFB1 and 0.4 mg/kg Ct-Se. The dosage of AFB1 and Ct-Se were referenced in our previously published literature (15, 17). The chicks were fed according to the commercial broiler diet standards recommended by the United States National Research Council (NRC) (18). Broilers were reared in environmentally controlled pens for a total of 42 days (Figure 1) under standardized environmental conditions as we previously described (19). Briefly, the ambient temperature was gradually decreased from 33 °C to 24 °C, and the relative humidity was maintained at 55–65%. The fluorescent lighting schedule was set to a 23 L: 1D cycle during the first week, and subsequently adjusted to an 18 L: 6D cycle. During the experimental period, individual body weight (BW) was recorded weekly. Average daily gain (ADG), average daily feed intake (ADFI), and feed conversion ratio (FCR; feed/gain) were calculated based on BW changes and feed consumption over the corresponding periods (19). At 42 days of age, the peripheral blood of broilers was collected via wing vein puncture prior to humane euthanasia (150 mg/kg sodium pentobarbital for intravenous injection). Blood samples were centrifuged to obtain serum and stored at −20 °C for subsequent biochemical and immunological assays. To determine the immune organ indices, the spleen, thymus, and bursa of Fabricius were collected, rinsed in ice-cold saline, blotted dry, and immediately weighed. One half of the spleen was fixed in 4% paraformaldehyde for histological and immunohistochemical analyses, whereas the other half was snap-frozen in liquid nitrogen and stored at −80 °C for further analyses.

### 2.2 Immune organ index calculation

At 42 days of age, broilers were randomly selected from each experimental group to measure the immunological indices (*n* = 6). The spleen, bursa of Fabricius, and thymus were cleared of adhering fat and connective tissue and weighed. The relative organ immune index was calculated as organ weight/body weight (g/kg) following the methods used in our previously published study (19).

### 2.3 Enzyme-linked immuno sorbent assay (ELISA) detecting

At 42 days of age, broilers were randomly selected from each experimental group to measure the serum immunoglobulin levels (*n* = 6). Blood samples were collected and centrifuged to obtain serum, and spleen was homogenized on ice and centrifuged to collect the supernatants. Serum immunoglobulin levels of IgA, IgG, and IgM were quantified using commercial ELISA kits (Shanghai Jonlnbio Industrial Co., Ltd., China). Similarly, the splenic levels of inflammatory cytokines, including IL-1β, IL-4, IL-6 and IFN-*γ*, were quantified using assay kits (Shanghai Enzyme-Linked Biotechnology Co., Ltd., China). All procedures were performed according to the manufacturers’ instructions. The optical density of each sample was recorded at 450 nm using a microplate reader (Synergy H1, BioTek Instruments, Inc., Winooski, USA), and the concentrations were calculated against standard curves.

### 2.4 Antioxidant and oxidative stress marker measurements

Spleen tissue was homogenized on ice, and the harvested supernatants were used to determine levels of malondialdehyde (MDA), total superoxide dismutase (SOD), total antioxidant capacity (T-AOC), and glutathione peroxidase (GSH-Px). These assays were performed using commercial kits according to the manufacturers’ instructions (Nanjing Jiancheng Bioengineering Institute, China). The optical density was recorded at specific wavelengths using a microplate reader (Synergy H1).

### 2.5 Histopathological examination

At 42 days of age, broilers were randomly selected from each experimental group, and spleen tissues were harvested for hematoxylin and eosin (H&E) staining (*n* = 3). Histopathological examination was performed as previously described (20). Briefly, after fixation, spleen tissue was dehydrated with graded ethanol series, cleared in xylene, infiltrated with melted paraffin, and embedded into paraffin. Sections (5 μm) were stained with hematoxylin and eosin (H&E, Wuhan Servicebio Technology Co., Ltd., China). The staining procedure followed standard protocols including deparaffinization, rehydration, nuclear and cytoplasmic staining, dehydration, clearing. Finally, the slides were coverslipped and examined under a microscope (Nikon E100, Nikon Corporation, Tokyo, Japan).

### 2.6 Terminal-deoxynucleotidyl transferase mediated dUTP nick-end labeling (TUNEL) analyses

Splenic apoptosis was detected via the TUNEL assay. Briefly, sections were treated with proteinase K, and subsequently stained using a one-step TUNEL apoptosis assay kit (Jiangsu KeyGen Biotech Co., Ltd., China) according to the manufacturer’s instructions. After washing, apoptotic nuclei were observed under a fluorescence microscope. The apoptotic index was calculated as following: (number of TUNEL-positive nuclei/total number of DAPI-stained nuclei) × 100%. For each section, TUNEL-positive cells were counted in three randomly selected high-magnification fields to obtain a representative apoptotic rate.

### 2.7 Western blot analysis

Total protein was extracted from spleen tissue using RIPA lysis buffer containing 1% protease inhibitors. Total protein concentration was determined using a BCA assay kit according to the manufacturer’s instructions. Equal amounts of protein were resolved on 10–15% SDS-PAGE gels and electrophoresed at 80 V for 30 min followed by 120 V for 50 min under standard denaturing conditions. Subsequently, separated proteins were transferred onto PVDF membranes by wet transfer at 200 mA for 50 min. After blocking with 5% skim milk in TBST, the membranes were incubated with the appropriate primary antibodies (Supplementary Table S1) at 4 °C overnight. Following three washes, membranes were incubated with horseradish peroxidase-conjugated secondary antibodies at room temperature for 1.5 h. Finally, bands were visualized using enhanced chemiluminescence reagents and captured with a JP-K4600 (Shanghai Jiapeng Technology Co., Ltd., China). The band intensity was analyzed using ImageJ software, with *β*-actin serving as the loading control.

### 2.8 Statistical analysis

All data are presented as mean ± standard deviation (SD). Statistical analyses were performed using GraphPad Prism 9.0 software (San Diego, USA). Two-way ANOVA was used as the statistical method, and Tukey’s test was used for *post-hoc* analysis. A *p* value < 0.05 was considered statistically significant.

## 3 Results

### 3.1 Ct-se alleviated AFB1-induced growth performance impairment in broilers

**Figure 2:**
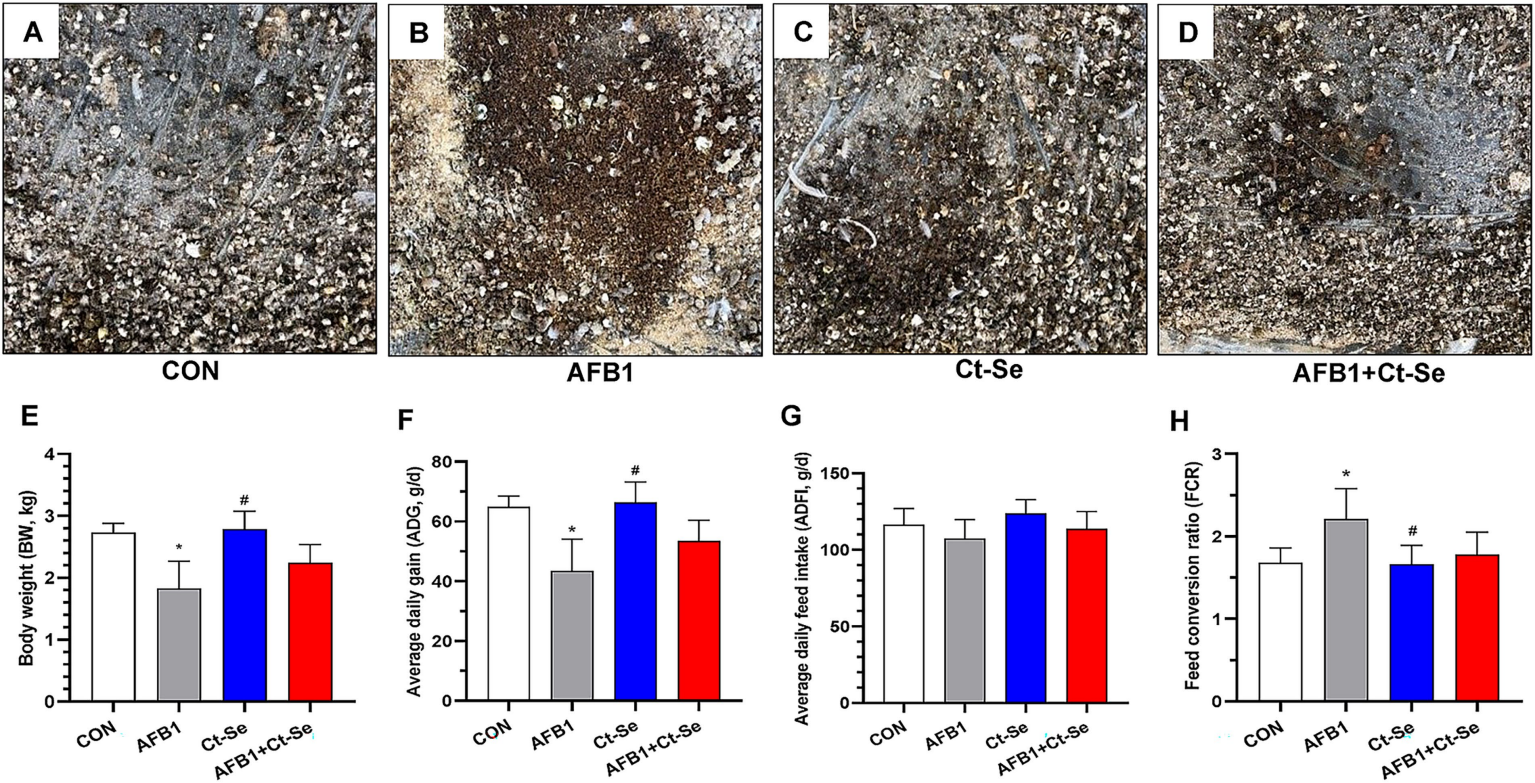
Ct-Se alleviates AFB1-induced alterations in growth performance of broilers. **(A–D)** Representative fecal appearance of broilers at 21 days of age in different treatment groups. **(E–H)** Body weight (BW), average daily gain (ADG), average daily feed intake (ADFI), and feed conversion ratio (FCR) of broilers during the experimental period in different groups. Data are presented as mean ± SD (*n* = 12). * p < 0.05 indicates significant difference compared to the CON group; ## *p* < 0.01 < # *p* < 0.05 indicates significant difference compared to the AFB1 group.

During the trial, the experimental groups showed distinct differences in clinical signs and growth performance. At 14 days of age, broilers in the CON, Ct-Se, and AFB1 + Ct-Se groups exhibited normal fecal morphology, characterized by well-formed strip- or pellet-shaped feces with small amounts of white urates and no obvious foul odor (Figures 2A,C,D). In contrast, broilers in the AFB1 group showed apparent feed passage, with soft, poorly formed feces accompanied by a strong foul odor (Figure 2B). Moreover, from 21 days of age onward, some broilers in the AFB1 group exhibited drooping wings and feather loss (data not shown). The results of growth performance are illustrated in Figures 2E–H. There were no significant differences in the ADFI among the groups. Compared with the CON group, the BW and ADG of broilers in the AFB1 group were significantly reduced by 33.1% (*p* < 0.05) and 32.9% (*p* < 0.05), respectively. This was accompanied by a 23.9% significant increase in FCR (*p* < 0.05). Compared with the AFB1 group, dietary supplementation with Ct-Se significantly increased ADG by 34.5% (*p* < 0.05) and reduced FCR by 24.9% (*p* < 0.01). However, no significant differences in BW, ADG, or FCR were observed between the AFB1 + Ct-Se group and the CON group (*p* > 0.05). Collectively, these results indicate that Ct-Se supplementation alleviates AFB1-induced growth performance impairment in broilers.

### 3.2 Ct-se ameliorated AFB1-induced reduction in immune organ indices and serum immunoglobulins

**Figure 3:**
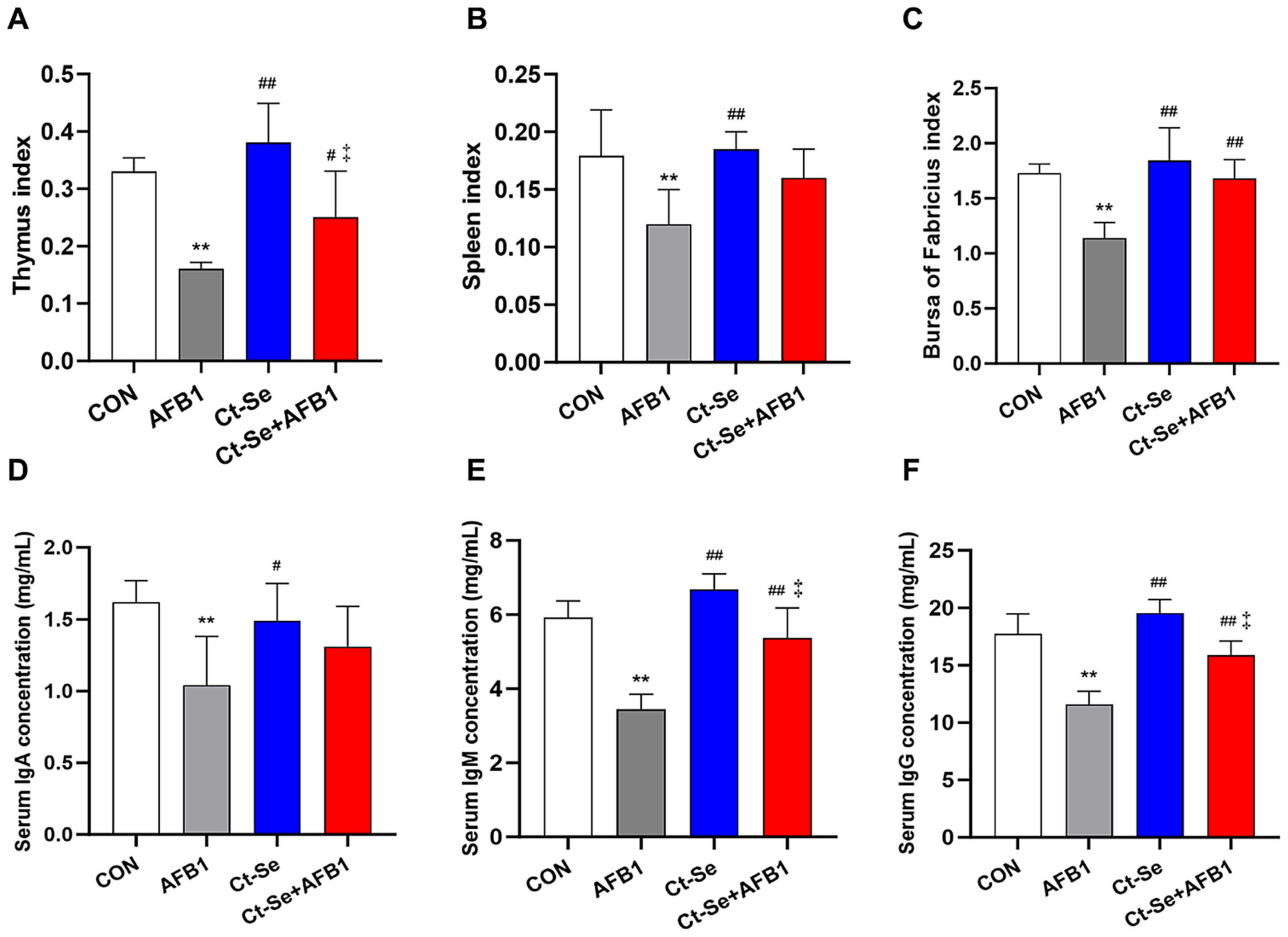
Ct-Se ameliorated AFB1-induced reduction in immune organ indices and serum immunoglobulins. **(A–C)** Relative weights of the thymus, spleen, and bursa of Fabricius at 42 days of age were evaluated and analyzed among groups. **(D–F)** Serum IgA, IgM and IgG levels in 42-day-old broilers were determined using commercially assay kits following the manufacturers’ protocols. Data are expressed as the mean ± SD (*n* = 6). ** p < 0.01 < * p < 0.05 indicates significant difference compared to the CON group; ## *p* < 0.01 < # *p* < 0.05 indicates significant difference compared to the AFB1 group; ‡ *p* < 0.01 < † *p* < 0.05 indicates significant difference between the Ct-Se group and the AFB1 + Ct-Se group.

The immunological indices of broilers at 42 days of age are shown in Figures 3A–C. The thymus, spleen, and bursa of Fabricius indices in the AFB1 group were significantly decreased than those in the CON group (all *p* < 0.01) and Ct-Se group (all *p* < 0.01). In contrast, there was no significant difference in the thymus, spleen, and bursa of Fabricius indices between the Ct-Se group and the CON group (*p* > 0.05), indicating that Ct-Se alone did not adversely affect immune organ development. Notably, the thymus indices (*p* < 0.05) and bursa of Fabricius indices (*p* < 0.01) in the AFB1 + Ct-Se group were significantly increased than those in the AFB1 group. This indicates that Ct-Se effectively mitigated AFB1-induced reductions in immunological indices. To evaluate the protective effect of Ct-Se against AFB1-induced immune injury in broilers, we measured the serum immunoglobulin levels (Figures 3D–F). The results showed that the contents of IgA, IgM, and IgG in the AFB1 group were significantly reduced in the CON group (all *p* < 0.01) and Ct-Se group (all *p* < 0.01). No significant differences were observed in the contents of IgA, IgM, and IgG between the Ct-Se group and the CON group (*p* > 0.05). However, the concentrations of IgM and IgG in the AFB1 + Ct-Se group were significantly higher than those in the AFB1 group (both *p* < 0.01), although they remained significantly lower than those in the Ct-Se group (both *p* < 0.01). These results indicates that Ct-Se could alleviate AFB1-induced immunosuppression in broilers.

### 3.3 Ct-se attenuated AFB1-induced oxidative stress and inflammatory damage in spleen tissue

**Figure 4:**
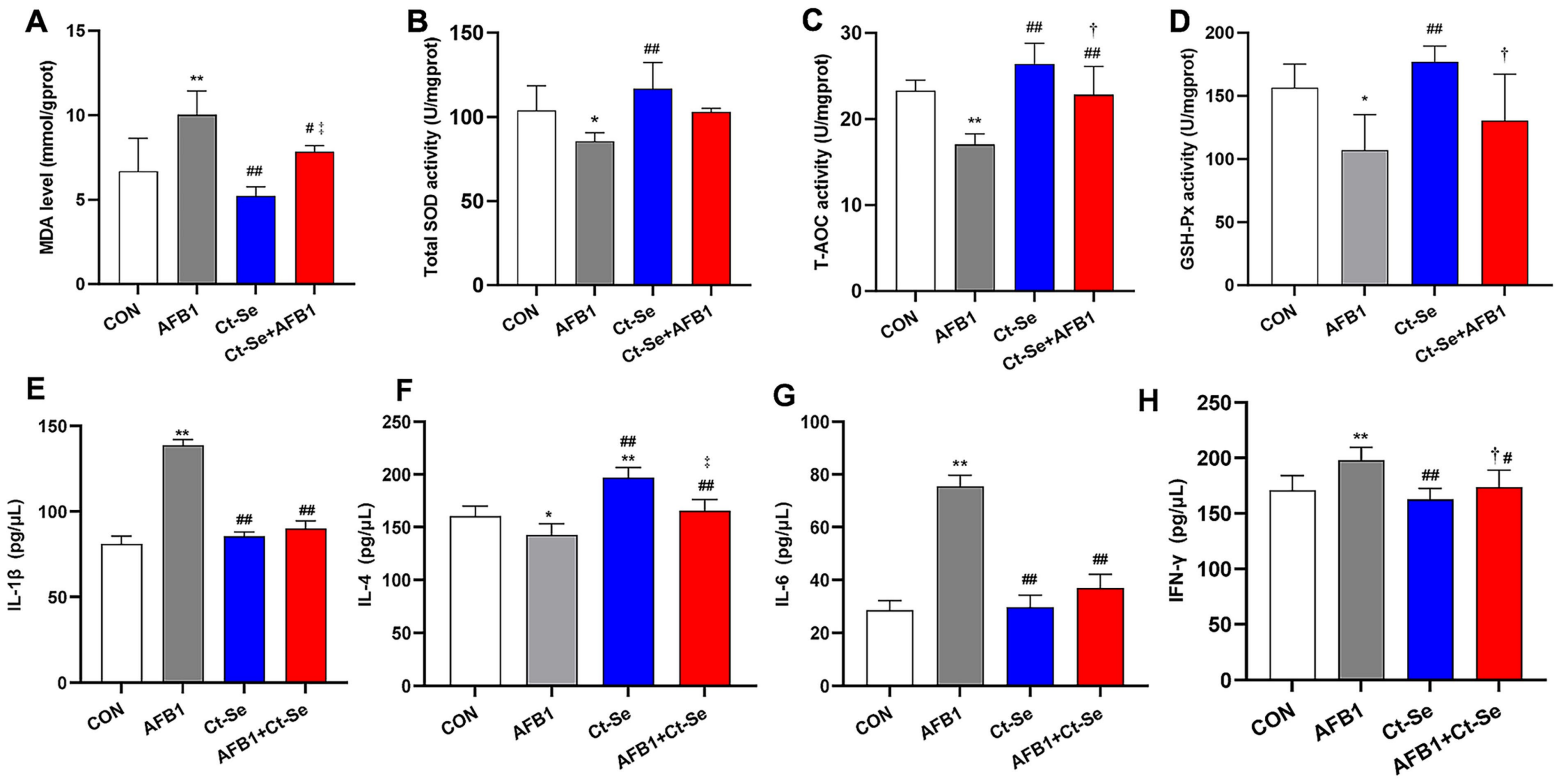
Ct-Se attenuated AFB1-induced oxidative stress and inflammatory response in spleen tissue. **(A-D)** MDA content and SOD, T-AOC and GSH-Px activities were determined using commercially assay kits following the manufacturers’ protocols. **(E–H)** Inflammatory cytokines in spleen tissue from different treatment groups were determined using commercially assay kits following the manufacturers’ protocols. Data are expressed as the mean ± SD (*n* = 6). ** p < 0.01 < * p < 0.05 indicates significant difference compared to the CON group; ## *p* < 0.01 < # *p* < 0.05 indicates significant difference compared to the AFB1 group; ‡ *p* < 0.01 < † *p* < 0.05 indicates significant difference between the Ct-Se group and the AFB1 + Ct-Se group.

To further validate the protective effect of Ct-Se against AFB1-induced immune injury in broilers, splenic oxidative stress and inflammatory parameters were assessed. As illustrated in Figures 4A–D, the MDA content in the AFB1 group was markedly elevated compared with the CON group (*p* < 0.01). However, the activities of antioxidant enzymes, including SOD (*p* < 0.05), T-AOC (*p* < 0.01), and GSH-Px (*p* < 0.05), were significantly reduced in the AFB1 group compared with the CON group. There were no significant differences in the levels of MDA, SOD, T-AOC, and GSH-Px between the Ct-Se group and the CON group (*p* > 0.05). Importantly, compared to the AFB1 group, the MDA content was significantly decreased, and T-AOC activity was significantly increased in the AFB1 + Ct-Se group (*p* < 0.01). These findings indicate that AFB1 induces oxidative stress damage, whereas Ct-Se effectively mitigated this impairment in the broiler spleen. To further evaluate the protective effects of Ct-Se against AFB1-induced splenic injury, the levels of inflammatory cytokines were determined (Figures 4E–H). The levels of IL-1β, IL-6 and IFN-*γ* in the AFB1 group were significantly upregulated compared with the CON group (all *p* < 0.01), whereas the IL-4 level was significantly downregulated (*p* < 0.05). Compared with the AFB1 group, the levels of IL-1β (*p* < 0.01), IL-6 (*p* < 0.01) and IFN-γ (*p* < 0.05) in the AFB1 + Ct-Se group were significantly decreased, while IL-4 was significantly restored (*p* < 0.01). These results indicate that Ct-Se effectively mitigates AFB1-induced inflammatory damage in the broiler spleen.

### 3.4 Ct-se alleviated AFB1-induced histopathological damage in spleen of broilers

**Figure 5:**
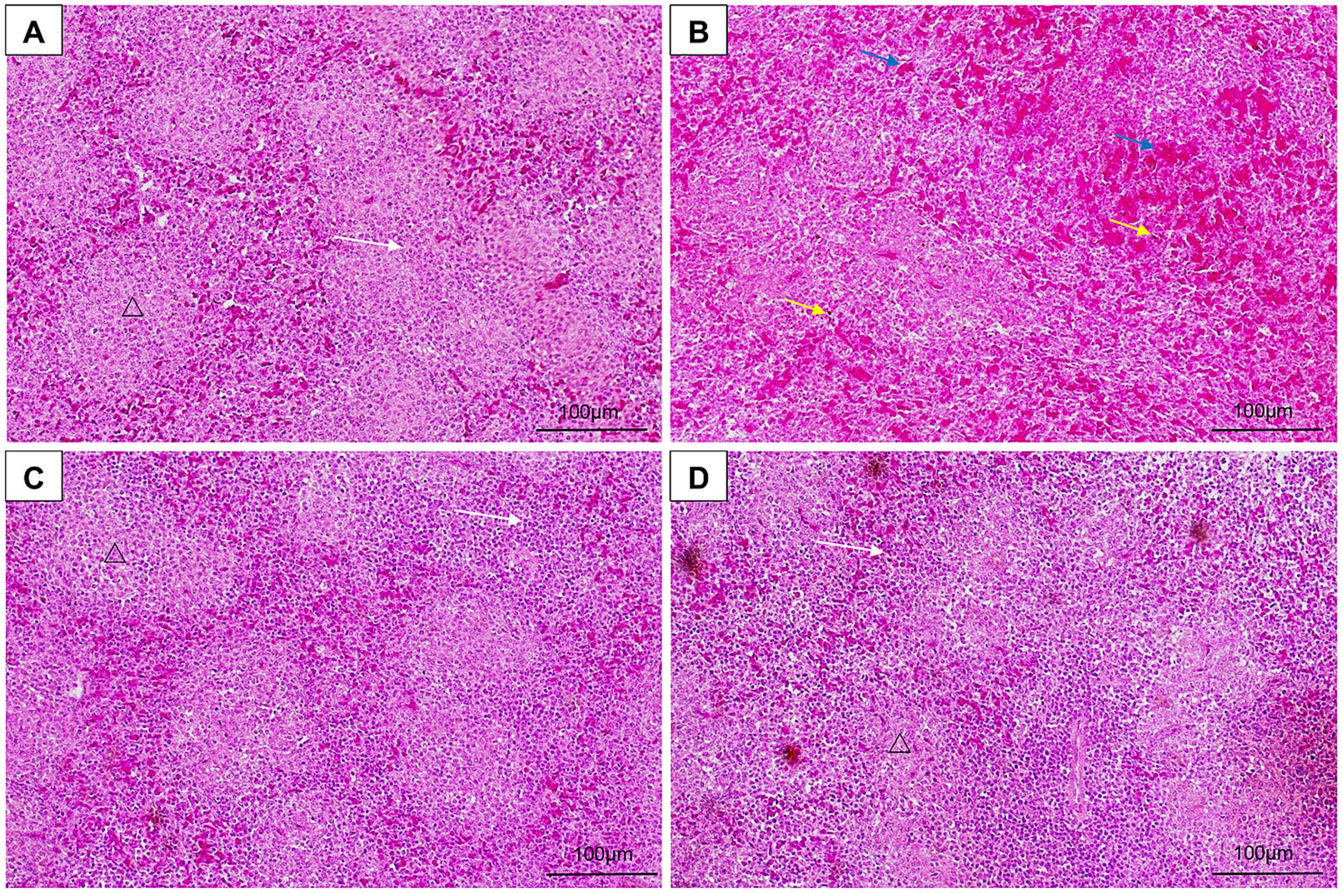
Ct-Se alleviated AFB1-induced histopathological damage in spleen. Histological examination of H&E staining of the spleen of broilers in the CON **(A)**, AFB1 **(B)**, Ct-Se **(C)**, and AFB1 + Ct-Se **(D)** groups, respectively, (*n* = 3). The scale bar represents 100 μm, △ indicates splenic nodules. In the AFB1 group, the boundaries between red and white pulp were indistinct, red pulp congestion (blue arrows), lymphocyte reduction (white arrows) and apoptosis (yellow arrows) were observed. The CON and Ct-Se groups maintained normal tissue structure, and co-treatment with Ct-Se notably improved histological architecture.

To intuitively evaluate the protective effect of Ct-Se against AFB1-induced splenic damage, histological sections at 42 days of age were evaluated. In the CON group and Ct-Se group (Figures 5A,C), the boundaries between red and white pulp were clearly defined and lymphocyte distribution appeared normal. In contrast, chickens in the AFB1 group displayed pronounced histopathological alterations, characterized by indistinct red-white pulp demarcation, severe red pulp congestion, decreased lymphocyte density, and noticeable vacuolization within lymphoid follicles (Figure 5B). However, in the AFB1 + Ct-Se group, the severity of pathological lesions was markedly reduced, evidenced by clearer red-white pulp boundaries, relieved congestion, and fewer vacuoles (Figure 5D). These findings demonstrate that Ct-Se effectively alleviate AFB1-induced structural disruption in the broiler spleen.

### 3.5 Ct-se attenuated AFB1-induced apoptosis in spleen of broilers

**Figure 6:**
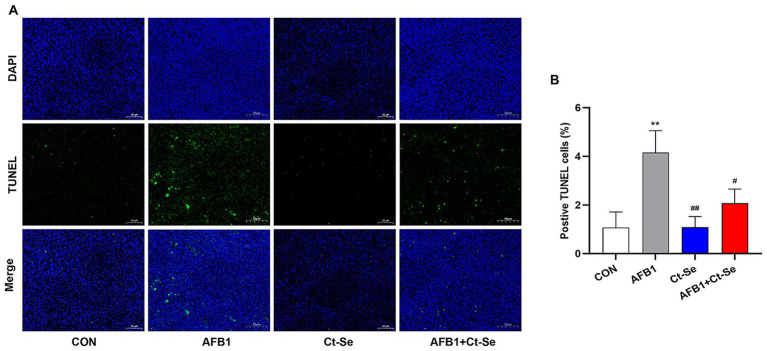
Ct-Se attenuated AFB1-induced apoptosis in spleen determined by TUNEL staining. **(A)** Representative the TUNEL staining results in spleen sections (*n* = 3). The TUNEL-positive cells were stained green, and the bars represent 50 μm. **(B)** Quantification of apoptosis index using Image J. The formula used for assessing the apoptosis rate was the number of TUNEL-positive nuclei (TUNEL specks) × 100%/the total number of nuclei (DAPI), and the data are expressed as mean ± SD (n = 6). ** p < 0.01 indicates significant difference compared to the CON group; ## *p* < 0.01 < # *p* < 0.05 indicates significant difference compared to the AFB1 group.

**Figure 7:**
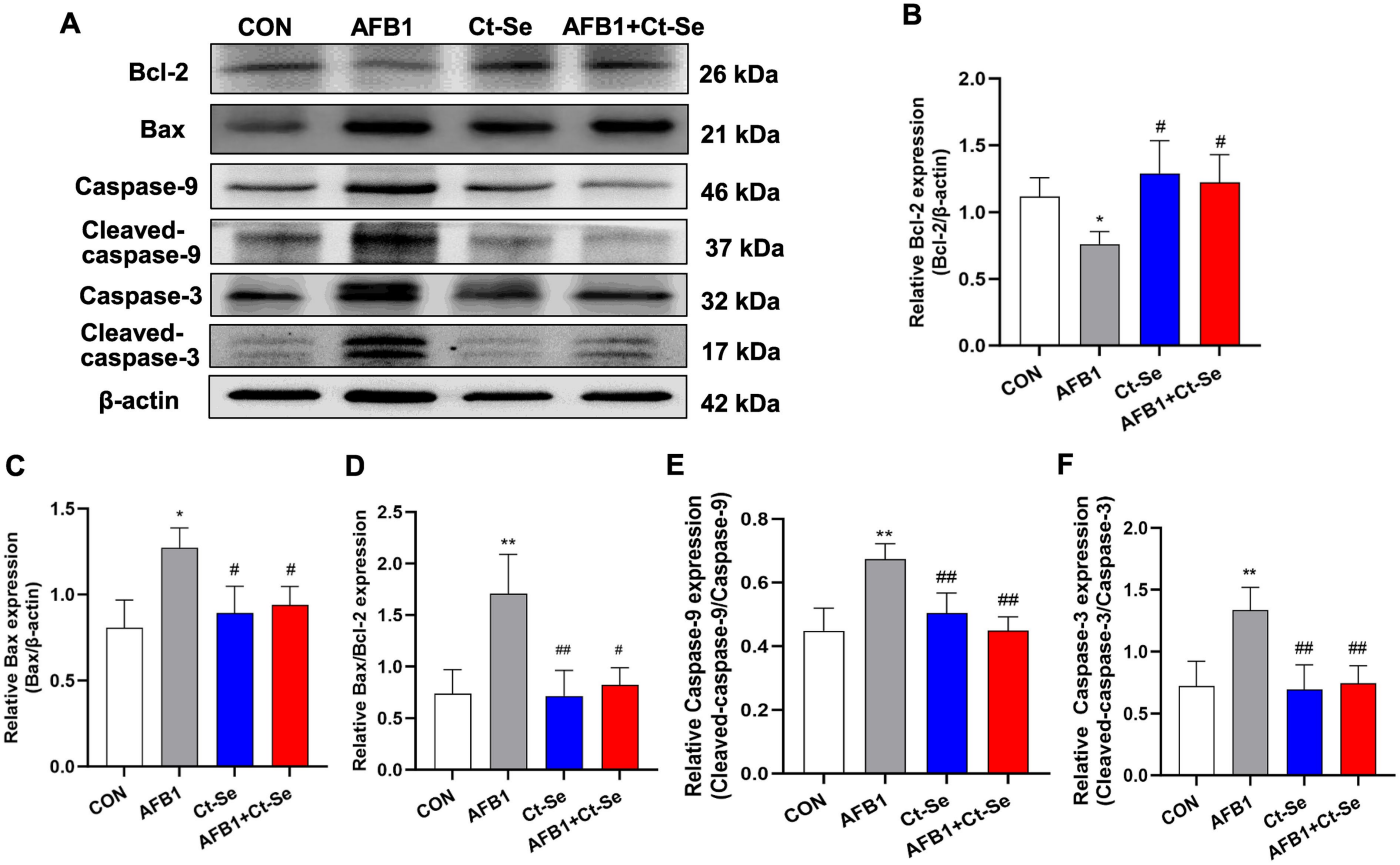
Ct-Se attenuated AFB1-induced apoptosis-related protein expression in spleen of broilers. **(A)** Western blot analysis of apoptosis-related proteins in spleen tissue. **(B–F)** Relative protein expression levels of Bcl-2, Bax, Bax/Bcl-2, cleaved-caspase-9, and cleaved-caspase-3. Data are presented as mean ± SD (*n* = 6). ** p < 0.01 < * p < 0.05 indicates significant difference compared to the CON group; ## *p* < 0.01 < # *p* < 0.05 indicates significant difference compared to the AFB1 group; ‡ *p* < 0.01 < † *p* < 0.05 indicates significant difference between the Ct-Se group and the AFB1 + Ct-Se group.

To further investigate the protective effect of Ct-Se against AFB1-induced splenic injury, the level of splenocyte apoptosis and the expression of key apoptosis-related proteins were determined. As the TUNEL staining results are shown in Figure 6, a marked elevation in the number of apoptotic splenocytes was observed in the AFB1 group compared with the CON group (*p* < 0.01). Conversely, the AFB1 + Ct-Se group exhibited a significant reduction in TUNEL-positive cells compare to the AFB1 group (*p* < 0.05), indicating that Ct-Se effectively alleviated AFB1-induced splenic apoptosis. The expression of spleen apoptosis-related proteins are shown in Figure 7. Consistent with the TUNEL results, AFB1 exposure significantly upregulated the pro-apoptotic proteins Bax (*p* < 0.05), cleaved-caspase 9 (*p* < 0.01) and cleaved-caspase-3 (*p* < 0.01), and downregulated the anti-apoptotic protein Bcl-2 (*p* < 0.05) compared to the CON group. These alterations resulted in a significantly elevated Bax/Bcl-2 ratio (*p* < 0.01, Figure 7D). No significant differences in the expression of these proteins were observed between the Ct-Se group and the CON group (all *p* > 0.05). Conversely, compared with the AFB1 group, the AFB1 + Ct-Se group exhibited significantly decreased expression of Bax (*p* < 0.05), cleaved-caspase-9 (*p* < 0.01), and cleaved-caspase-3 (*p* < 0.01), alongside increased Bcl-2 expression (*p* < 0.05), which ultimately significantly reduced the Bax/Bcl-2 ratio (*p* < 0.05). Together, these findings demonstrate that Ct-Se attenuates AFB1-induced splenic apoptosis in broiler.

## 4 Discussion

Accumulating evidence indicates that AFB1-induced immunotoxicity represents an equally important contributor to compromised poultry health and productivity (21). As the largest peripheral immune organ, the spleen plays a pivotal role in immune surveillance, lymphocyte activation, and humoral immune regulation, whereby its structural and functional integrity are rendered highly susceptible to disruption by AFB1-induced immunotoxicity (6). Specifically, AFB1 has been reported to induce splenic atrophy, lymphocyte depletion, and impaired immune function in poultry (3, 8), highlighting the spleen as a critical site for evaluating AFB1-induced immunotoxicity. In the present study, dietary AFB1 markedly impaired splenic immune function, as evidenced by decreased immune organ indices and suppressed serum immunoglobulin levels. Histopathological examination also revealed that AFB1 induced splenic injury characterized by red pulp congestion, lymphocyte depletion, and disorganization of the marginal zone. However, Ct-Se supplementation substantially ameliorated these splenic structural abnormalities and enhanced immune indices, immunoglobulin levels, and production performance, thereby providing direct evidence for the protective efficacy of this organic selenium in poultry against AFB1-induced immunotoxicity.

AFB1-induced immune dysfunction is closely linked to oxidative stress in immune organs. AFB1 has been shown to induce lipid peroxidation, suppress antioxidant enzyme activities (such as SOD, T-AOC and GSH-Px), and decrease IgG and IgA concentrations in chickens, thereby impairing immune organ function and humoral immunity (22, 23). The oxidative imbalance observed in this study indicated that oxidative stress serves as a critical upstream mechanism driving AFB1-induced splenic immunotoxicity. Our previous studies have demonstrated that Ct-Se enhanced the production performance and antioxidant capacity in laying hens (14), and protected mice against zearalenone-induced oxidative and immune damage (15). In addition, chitosan and its derivatives were reported to directly scavenge ROS and protect immune organs from oxidative injury (24, 25). Consistent with these protective properties, the present study revealed that Ct-Se significantly restored splenic antioxidant enzyme activities (SOD, T-AOC and GSH-Px) in AFB1 exposed broilers. These findings indicate that Ct-Se effectively mitigates AFB1-induced oxidative damage, thereby restored splenic immune function.

AFB1-triggered immunotoxicity is closely associated with inflammatory dysregulation. Previous studies have demonstrated that AFB1 can induce the overproduction of pro-inflammatory cytokines in immune organs, while suppressing the secretion of anti-inflammatory cytokines such as IL-4, thereby disrupting local immune homeostasis (14, 26). Our results revealed that Ct-Se ameliorated these abnormal inflammatory cytokine profiles by downregulating pro-inflammatory cytokines (IL-1β, IL-6, and IFN-*γ*) and restoring the anti-inflammatory cytokine IL-4. This indicates that Ct-Se alleviated AFB1-induced splenic inflammation and re-established the immune homeostasis, thereby recovering both serum immunoglobulin levels and immune organ indices. Furthermore, oxidative stress serves as a critical trigger for the inflammatory response, as it causes excessive ROS accumulation, thereby activating inflammatory signaling pathways (27, 28). These inflammatory responses aggravate local oxidative stress and contribute to further ROS production, resulting in a vicious cycle that exacerbates splenic immunotoxicity (27, 29). These findings indicate that Ct-Se maintains splenic immune homeostasis by mitigating AFB1-induced oxidative stress and inflammation crosstalk.

Crucially, severe oxidative and inflammatory stress also serves as the trigger for the apoptotic cascade. In this study, TUNEL staining revealed that AFB1 markedly increased the apoptotic rate in the spleen, whereas dietary Ct-Se significantly reduced this effect, demonstrating the anti-apoptotic role of Ct-Se in AFB1-induced spleen damage. At the cellular level, excessive oxidative stress directly compromises mitochondrial membrane integrity, and depletes antioxidant enzymes, which exacerbates cellular vulnerability and initiates the mitochondrial apoptotic program (30, 31). Indeed, AFB1-induced apoptosis is primarily mediated through the mitochondrial apoptotic pathway (32–34). Our study confirmed that AFB1 increased the Bax/Bcl-2 ratio and triggered mitochondrial-dependent apoptosis in spleen, which is consistent with previous reports (32–35). Furthermore, the overproduction of pro-inflammatory cytokines also exacerbates splenic apoptosis through inflammatory-apoptotic crosstalk (36, 37). Pro-inflammatory cytokines trigger ROS-mediated mitochondrial dysfunction and the caspase-9/3 cascade, while simultaneously recruiting NF-κB and MAPK pathways to imbalance Bax/Bcl-2 ratios (38, 39). This coordinates a self-amplifying feedback loop between inflammation and apoptosis.

Previous research has demonstrated that selenium supplementation effectively protects against AFB1-induced renal cell apoptosis, cell-cycle arrest, and hepatocyte apoptosis in broilers (40, 41). Conversely, selenium deficiency has been shown to increase splenic cell apoptosis and aggravate AFB1-induced hepatic injury (6, 42). Additionally, selenium supplementation has also been shown to alleviate splenic oxidative damage in broilers by modulating the NF-κB pathways (43). In mammalian injury and diseases models, selenium effectively interrupts the vicious cycle between inflammatory cytokine overproduction and redox imbalance (44, 45). These findings underscore the critical role of selenium in protecting against AFB1-induced apoptosis. Overall, due to its higher bioavailability and enhanced antioxidant capacity as previously described (13, 14, 16), Ct-Se effectively bolsters antioxidant defenses, and normalizing inflammatory cytokine secretion. This dual action mitigates both the oxidative and inflammatory amplifiers, thereby completely suppressing the downstream apoptotic cascade.

## 5 Conclusion

In conclusion, our results demonstrate that Ct-Se alleviates AFB1-induced splenic immunotoxicity and growth performance impairment in broilers. This protective effect is mediated through an integrated mechanism involving the restoration of immune function, enhancement of antioxidant capacity, suppression of inflammatory responses and inhibition of apoptosis.

## Data Availability

The original contributions presented in the study are included in the article/Supplementary material, further inquiries can be directed to the corresponding authors.
