# Supplementary material for: Chitosan-selenium alleviates AFB1-induced growth impairment and immunotoxicity in broilers by suppressing splenic oxidative stress, inflammation and apoptosis
Source: Front Vet Sci. 2026 May 29;13:1859938. doi: 10.3389/fvets.2026.1859938 (PMC13259984; doi:10.3389/fvets.2026.1859938)
Supplement: Supplementary file 1 [file Table_1.docx]

**Table S1 The primary antibodies used in this study**

| Antibodies Names | Dilution multiple | Item No. | Manufacturers (Anti-rabbit) |
| --- | --- | --- | --- |
| β-actin | 1:3000 | GB15003 | Wuhan Servicebio Co., Ltd., Hubei, China |
| Bax | 1:1000 | GB15690 | Wuhan Servicebio Co., Ltd., Hubei, China |
| Bcl-2 | 1:500 | GB154380 | Wuhan Servicebio Co., Ltd., Hubei, China |
| Caspase-3 | 1:1000 | WL04004 | Wanleibio Co. Ltd., Liaoning, China |
| Cleaved-caspase-3 | 1:500 | 341034 | Chengdu Zen-Bioscience Co., Ltd., Sichuan, China |
| Caspase-9 | 1:1000 | WL03421 | Wanleibio Co., Ltd., Liaoning, China |
| Cleaved-caspase-9 | 1:500 | R381336 | Chengdu Zen-Bioscience Co., Ltd., Sichuan, China |
